# Determination of Actual Friction Factors in Metal Forming under Heavy Loaded Regimes Combining Experimental and Numerical Analysis

**DOI:** 10.3390/ma9090751

**Published:** 2016-09-01

**Authors:** Ana María Camacho, Mariano Veganzones, Juan Claver, Francisco Martín, Lorenzo Sevilla, Miguel Ángel Sebastián

**Affiliations:** 1 Department of Manufacturing Engineering, Universidad Nacional de Educación a Distancia (UNED), Madrid 28040, Spain; mveganzon6@alumno.uned.es (M.V.); jclaver@ind.uned.es (J.C.); msebastian@ind.uned.es (M.Á.S.); 2 Department of Civil, Material and Manufacturing Engineering, University of Malaga, Malaga 29071, Spain; fdmartin@uma.es (F.M.); lsevilla@uma.es (L.S.)

**Keywords:** friction, lubricant, FE, tribology, metal alloys, forming, large deformations, experiments

## Abstract

Tribological conditions can change drastically during heavy loaded regimes as experienced in metal forming; this is especially critical when lubrication can only be applied at the early stage of the process because the homogeneous lubricant layer can break along the die-workpiece interface. In these cases, adopting a constant friction factor for the lubricant-surface pair may not be a valid assumption. This paper presents a procedure based on the use of dual friction factor maps to determine friction factors employed in heavy loaded regimes. A finite element (FE) simulation is used to obtain the friction factor map for the alloy UNS A96082. Experiments were conducted using four lubricants (aluminum anti-size, MoS_2_ grease, silicone oil, and copper paste) to determine the actual friction curves. The experimental procedure is based on the application of lubricant only at the beginning of the first stage of ring compression, and not at intermediate stages as is usual in typical ring compression tests (RCTs). The results show that for small reductions (*r_h_* < 20%), the conventional RCT can be applied because the tribological conditions remain similar. For large reductions (*r_h_* > 20%), it is recommended to obtain an average value of the friction factor for every lubricant-surface pair in the range of deformation considered.

## 1. Introduction

Friction reduction has typically been investigated due to its importance in mechanical processes performance, systems performance, and wear prevention. An effective technique for improving tribological performance is surface texturing, as demonstrated by Lu et al. in [1] where friction reduction is achieved by generating square dimples of different sizes and geometries at the contact surface. New coatings to prevent wear under severe conditions are also being investigated, as observed in the work from Vandoni et al. [2], where the use of fiber laser sources for surface texturing of very thin TiN coatings is presented as a good option for heavy loaded sliding regimes. Reduction of friction and wear has been proposed in the recent work of Yazawa et al. [3] through a hybrid tribofilm consisting of both coating and lubricant. In metal forming, friction is a very complex phenomenon due to the variety of technological factors involved and their own interrelation. Not only friction reduction, but also friction characterization has grown recently because tribological conditions between workpieces and tools heavily influence material flow and tool life [4], as well as required loads, energy consumption, surface quality, and the internal microstructure of the products obtained by plastic deformation. Recent studies published by Hua et al. [5] and Zhang and Ou [6] show the enthusiastic interest of the scientific community to develop new methods for friction characterization and to discover new relationships between classical friction models, respectively. The most widely accepted methodology to characterize friction between two surfaces is to define a friction factor at the work piece-die interface. To evaluate the behavior of lubricants in metal forming processes, it is common to quantify the friction factor using different indirect techniques, such as the open-die backward extrusion test [7], double cup extrusion test [8], or ring compression test, among others. The ring compression test (RCT) has been widely used and successfully applied since its initial development by Kunogi [9]; this method was improved by the well-known work of Male and Cockcroft [10]. After its publication, many researchers have subsequently justified its validity; one example is the work of Hawkyard and Johnson [11] that analyzed the problem assuming homogeneous deformation, neglecting strain hardening, and assuming a constant Tresca friction factor at the interface. Male performed a study to determine the variations of the friction factor of metals during the compression processes at room temperature [12], and later on, the same author investigated the applicability of the RCT to conventional metal forming processes [13]. Carter and Lee [14] simulated the RCT using a finite element model, generating the friction factor curves for a particular material, and they observed some differences compared to the curves obtained by Male and Cockcroft in their 1965 work. These differences were related to the assumption of homogeneous strain and a constant value of the friction factor at the workpiece-die interface. Some years later Wang and Lenard [15] concluded that the temperature and strain rate were the two most influential parameters on the Tresca friction factor form results of hot RCTs. Other works have attempted to determine the dependence of friction on the material properties, strain rate, and non-homogeneous deformation, using both metallic [16] and non-metallic materials [17]. From these works it can be concluded that the RCT is an effective method for the determination of the friction factor in the metal forming processes. However, it is inadvisable to use generic friction maps and tables independent of the type of material and operating conditions, as demonstrated by several authors in the scientific literature [7,18]. Particularly, during any transient metal forming operation, such us forging, tribological conditions change during the process; this is especially critical when lubrication can only be supplied at the early stage of the process and heavily loaded conditions are applied due to the extreme changes during the forming process that occur to the lubricant layer at the interface. In these cases, the consideration of the initial friction factor for the lubricant-surface pairs applied to the entire operation can lead to an underestimation of the required forces and, consequently, of the required energy to finish the operation. To determine the actual friction factors found during these operating conditions, numerical simulation is required as a complementary analysis tool to experiments. The Finite Element Method (FEM) is widely used in metal forming simulation because its analysis capability has been probed under large deformations. The combination of experimental tests with analytical and numerical techniques has become the most powerful methodology for researchers in metal forming. As an example, Shahriari et al. [19] have studied the hot RCT of the superalloy Nimonic 115 by combining simulation techniques and experimental tests while using a profile projector as a method of measurement of ring dimensions. Zhu et al. [20] also determined the friction factor of Ti-6Al-4V titanium alloy in hot forging by combining RCT results and finite element simulations. Other analytical techniques such as the Upper Bound Theorem (UBT) also have considerable potential, as demonstrated by Bermudo et al. in their recent work [21]. This paper presents an alternative use of the RCT (by means of dual friction factor maps) to determine friction factors more adapted to heavy loaded regimes that occur during metal forming by combining experiments with numerical simulations by FEM.

## 2. Materials and Methods

### 2.1. Approach

**Figure 1:**
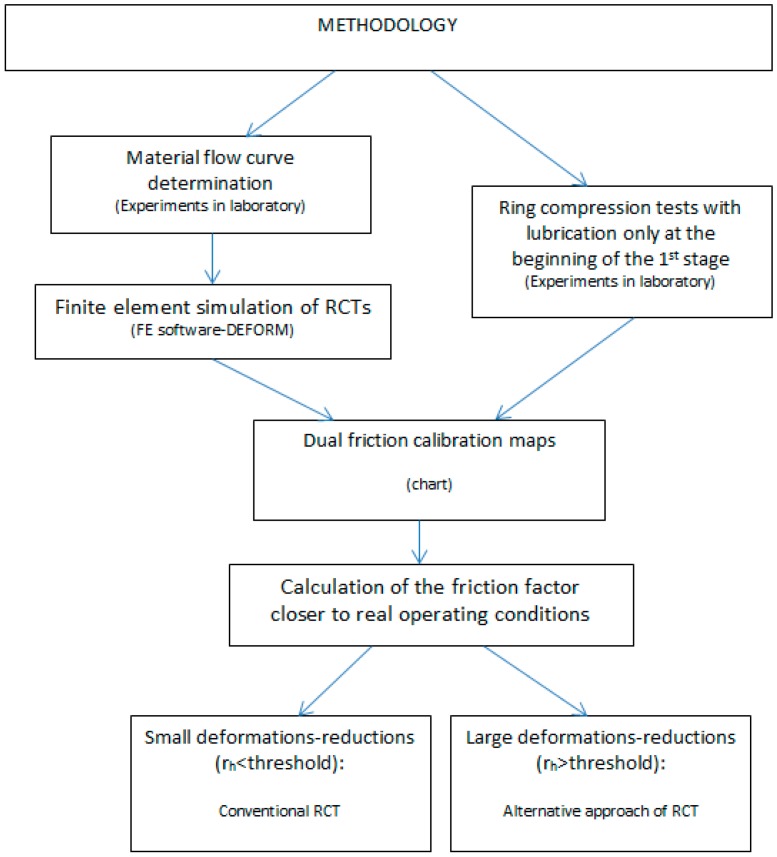
Outline of the methodology. RCT refers to ring compression test.

In this work, a combination of experiments with numerical simulations by the FEM has been selected to perform the analysis and the procedure followed is outlined in Figure 1.

This methodology will be explained in detail hereafter, and it comprises the following:

- Experimental determination of the plastic flow curve for the material UNS A96082 used in the RCTs.
- Finite Element modelling to simulate RCTs, considering the flow curve previously obtained.
- Experimental performance of RCTs according to the alternative procedure presented in this paper. This procedure is based on the application of lubricant only at the beginning of the first stage during the compression of the rings and not at intermediate stages as usual in typical RCTs.
- Graphical representation of the dual friction factor map, which includes both the friction curves obtained by experiments in laboratory and by numerical simulations.
- Selection of the friction factor according to the deformation stage.

### 2.2. Determination of the Plastic Flow Curve

Before starting the numerical simulation, the flow curve of the material UNS A96082 (Sanmetal SA, Zaragoza, Spain) used in the experimental tests had to be determined. The Bulge Correction Factor Method (BCFM) [22] was used to obtain the plastic flow curve of the aluminum alloy UNS A96082 by means of compression tests under controlled conditions in the laboratory. During uniaxial compression tests, the specimen undergoes plastic deformation once the yield stress is achieved, after the elastic regime [23]. In the plastic regime, there is a uniform deformation at first; afterwards, the friction at the workpiece-compression dies’ interfaces causes a non-uniform deformation, so a correction factor is required to calculate the flow stress in correctly [24].

**Figure 2:**
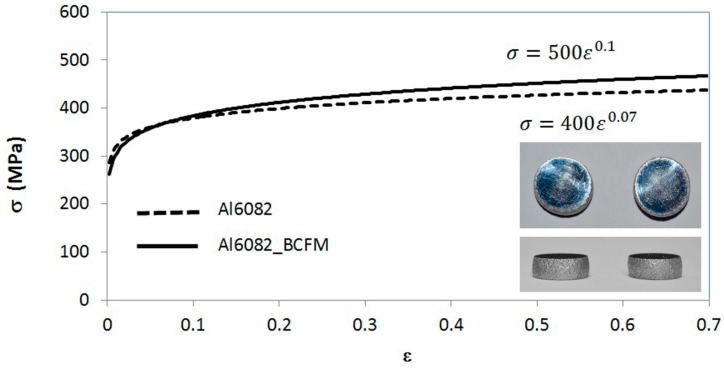
Plastic flow curve of the Aluminum alloy UNS A96082.

To accomplish this goal, controlled forming under quasi-non-friction conditions was performed in the laboratory. During the compression of each specimen, appropriate measurements were gathered to obtain the flow curve according to the BCFM. In Figure 2, both curves (before and after application of BCFM) are represented.

To characterize the material and to implement its plastic properties into the finite element model, the flow curve according to Hollomon’s expression is presented (Equation (1)):

σ (MPa) = 500·ε^0.1^ (1)

where *K* = 500 MPa is the strength coefficient and *n* = 0.1 is the strain hardening exponent. They have been determined as a result of potential fitting of the data obtained in the plastic zone of the stress-strain curve by uniaxial compression tests of cylindrical specimens.

### 2.3. Lubricants

Lately, there is a growing interest in the use of green lubricants due to environmental concerns [25,26]. Lubricants are commonly applied in the form of liquid or solid films at the die-workpiece interface to minimize adhesion, the interaction between surfaces and, therefore, friction [27]. The most common lubricants in use are oils and greases. Oils are basically composed of a base oil and specific additives. These additives are added to the base oil to provide the lubricant its properties and performance characteristics. On the other hand, grease comprises a base oil, additives, and a thickener. The thickener may be any material that, in combination with the base oil, will produce the solid to semi-fluid structure. The main thickeners used in greases include lithium, aluminum, calcium soaps, and clay, either alone or in combination. Lithium soap is the most common thickener in use today. However, many applications use solid lubricants as in the case of molybdenum disulphide (MoS_2_), boron nitride, polytetrafluoroethylene (PTFE) or Teflon, and graphite. These are mainly used in warm and hot forming where liquid lubricants are not recommended. These lubricants are widely used in the metal-mechanical industry [28].

To simulate different friction conditions during experimental tests, four lubricants (namely aluminum anti-size, MoS_2_, silicone oil, and copper paste) were used and characterized by means of the RCT. Aluminum anti-size has excellent lubricating, anticorrosive, and anti-seize properties due to the aluminum structure that provides excellent behavior in high temperature conditions (until 600 °C). It is recommended to prevent early wear of surfaces. MoS_2_ grease contains a mineral oil as a base, an organic thickener, and additives for use in high pressure conditions; it is widely used in cases where oscillations, vibrations, and impact loads are encountered under moderate temperatures. Silicone oil can be used for metallic and non-metallic components; it is characterized by a low viscosity level and a high resistance against decomposition by heat. The last lubricant is the copper paste; it is typically used to prevent wear by corrosion in high temperature and high load applications.

### 2.4. Finite Element Model

A finite element model has been developed in DEFORM F2™ (Scientific Forming Technologies Corporation, Columbus, OH, USA) to accomplish the goal of creating a friction factor map for the specific material UNS A96082. This FE code is a computer aided engineering software specifically designed for metal forming analysis. The DEFORM (Scientific Forming Technologies Corporation, Columbus, OH, USA) preprocessor uses a graphical user interface to assemble the data required to run the simulation. Input data includes:

- Object description: all data associated with an object, including geometry, mesh, temperature, material, etc.
- Material data: data describing the behavior of the material under the conditions which it will reasonably experience during deformation.
- Inter object conditions: describes how the objects interact with each other, including contact and friction between objects.
- Simulation controls: definition of parameters such as discrete time steps to model the process.

The main concepts of the preprocessing stage are explained in detail hereafter.

**Figure 3:**
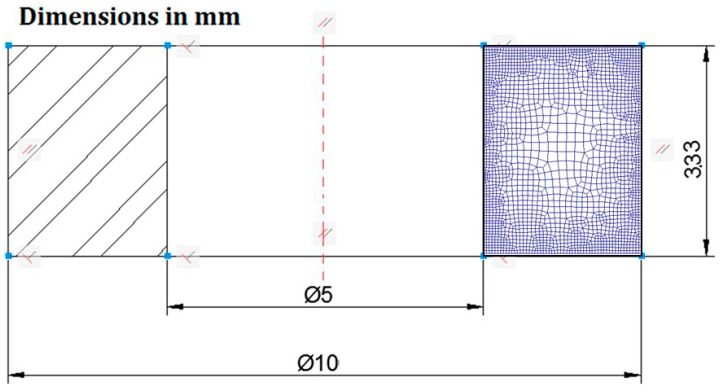
Geometry of the rings tested by RCT.

The geometrical relationships of the dimensions of the rings and the operating parameters involved in the process were based on those used by Sofuoglu and Rasty in their tests [17]. The most common dimensional ratio used in this type of problem is the relation between outer diameter: inner diameter: height, 6:3:2, respectively, commonly named the “canonical aspect ratio”. Specific dimensions used in the rings tested are shown in Figure 3.

Symmetry conditions are typically applied in the literature about RCTs due to the axisymmetric nature of this problem. This can be observed in the finite element modelling of RCT in works of reference such as the ones realized by Sofuoglu et al., where bi-dimensional models using finite element codes ANSYS [7] and Abaqus [16,17] have been developed. Considering this, an axisymmetric model was created with the finite element software DEFORM F2™ (Scientific Forming Technologies Corporation, Columbus, OH, USA); symmetry conditions were imposed, so only half of the model was analyzed.

Flat platens are modelled as rigid parts and the workpiece is modelled as a deformable body. The workpiece has been meshed with first order continuum elements of quadrilateral shape and the mesh contains approximately 2200 elements.

Regarding the material, each ring has been modelled with aluminum alloy UNS A96082, whose plastic flow curve was determined previously from the compression test, as explained above. The flow stress data are introduced as tabular data because this is the most highly recommended method to follow the true behavior of the material:

(2) $$\bar{\sigma }=\bar{\sigma }\left(\bar{\varepsilon },\bar{\dot{\varepsilon }},T\right)$$

where

$\bar{\sigma }$: effective flow stress,
$\bar{\varepsilon }$: effective plastic strain,
$\bar{\dot{\varepsilon }}$: effective strain rate,
T: work temperature

In this paper cold forming conditions are considered, so the flow stress does not depend on the strain rate as the temperature is considered constant and equal to 20 °C.

A constant friction model or shear friction model was assumed for a range of friction factors, “*m*”, from 0 to 0.6. This friction model considers a friction factor, *m*, to quantify the interface friction and its analytical expression is (Equation (3)):

(3) τ=m⋅k

This model assumes that friction stress is constant and it only depends on the shear flow stress, *k*. For example, for perfect lubrication (*m* = 0) friction stress is null, whereas for sticking conditions (*m* = 1), friction stress equals the shear flow stress. This model has been demonstrated to be more realistic than Coulomb’s friction model in metal forming analysis because normal pressures are often higher than flow stresses so the Coulomb’s friction model provides friction stresses higher than shear flow stresses.

Regarding simulation controls, DEFORM F2™ is a numerical code of implicit methodology that uses the Newton-Raphson method for solving the equations. The model includes 200 steps and the step increment is defined as 10. The number of steps is given by Equation (4):

(4) $$n=\frac{x}{V\cdot \Delta t}$$

where

*n*: number of steps,
*x*: total movement of the primary die,
*V*: primary die velocity,
Δ*t*: is the time increment per step

**Figure 4:**
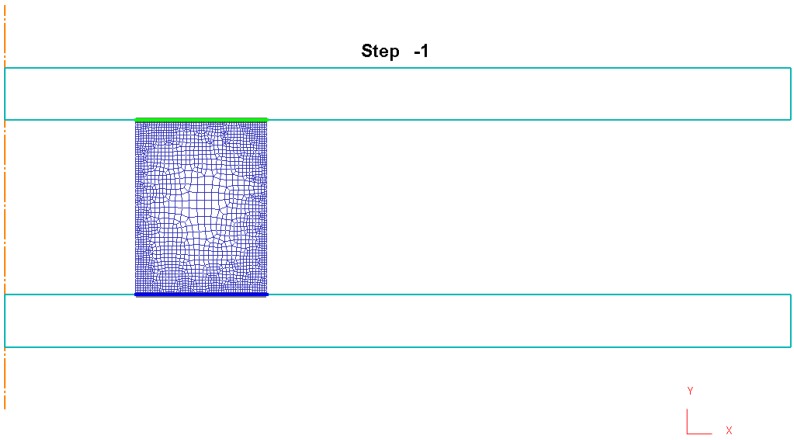
Finite Element model by DEFORM F2™ after preprocessing.

The completed model after preprocessing is presented in Figure 4, where contact nodes at the beginning of the compression process are shown at the die-workpiece interfaces.

**Figure 5:**
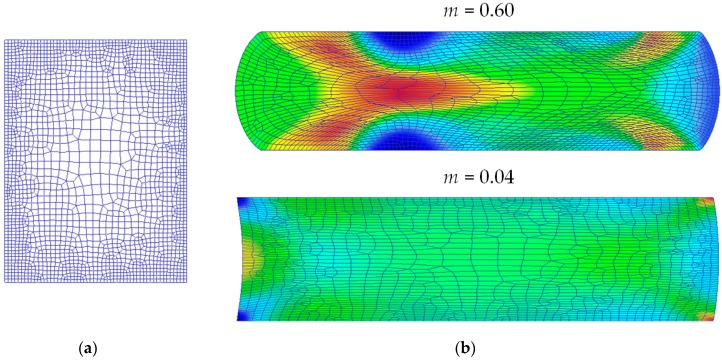
Finite Element Method (FEM) simulation of RCT for different friction conditions. (**a**) Initial mesh (axisymmetrical model); (**b**) Deformed samples for different friction conditions (top: *m* = 0.6; bottom: *m* = 0.04).

The deformed rings after compression and extreme friction conditions are shown in Figure 5b for comparison. As explained in [18], when a ring preform is compressed in the plastic field between flat platens, a high friction factor results in an inward flow of material (*m* = 0.6); however, a low friction factor results in an outward flow of material (*m* = 0.04).

### 2.5. Experimental Procedure

**Figure 6:**
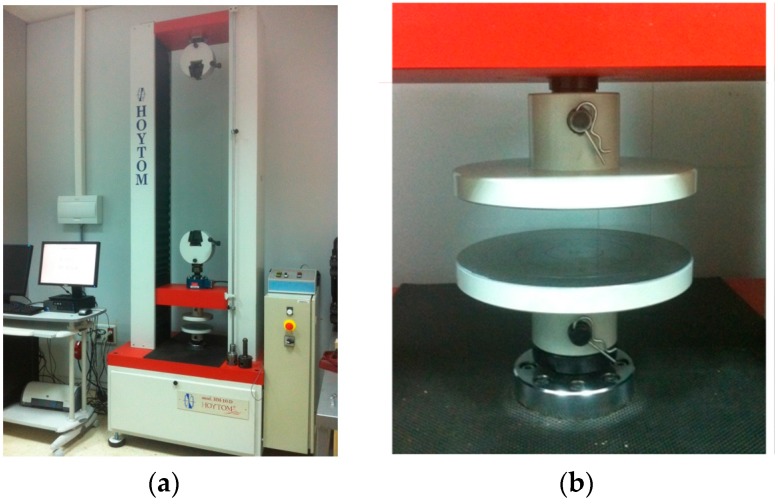
Equipment and forming tools. (**a**) Universal test machine HOYTOM HM-100kN; (**b**) Detailed view of flat platens.

Experimental tests were conducted to determine the dual friction factor map of the aforementioned lubricants for material UNS A96082 under specific forming conditions. Accordingly, experimental compression tests were realized using rings treated with the lubricants presented; for this purpose, the universal test machine HOYTOM HM-100kN (HOYTOM S.L., Leioa, Spain) (Figure 6a) and flat platens as forming tools (Figure 6b) were used.

To conduct the RCTs, each ring was placed on the bottom plate of the test machine (compression area) after lubrication of the contact surfaces; then, the top plate was positioned making contact with the upper surface of the ring. Once contact was made, an increment of the load was applied causing a reduction in height and the deformation of the inner radius. According to the alternative approach presented in this paper, the lubricant was only applied at the first stage, as explained previously.

The RCTs were conducted on three different rings for each lubricant in order to generate enough points to create the friction map. Compressions were applied to reach loads of 30, 50, and 70 kN. The ram velocity of the upper plate of the test machine was 2.5 mm/s in all of the cases. The conditions of the process are specified in Table 1.

**Figure 7:**
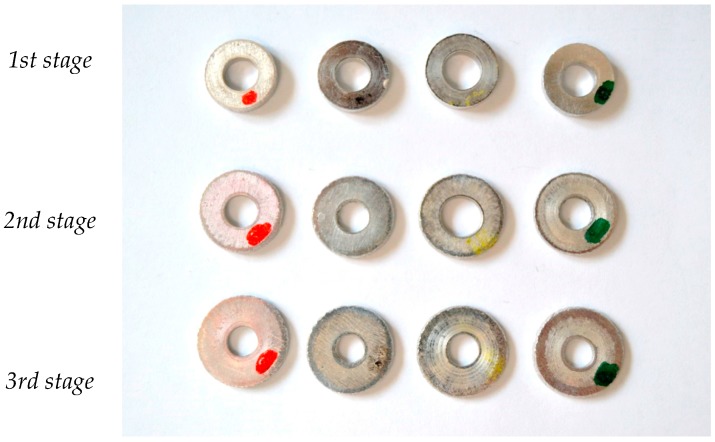
Rings after each compression stage using four lubricants. From left to right: copper paste, silicone oil, MoS_2_ grease, aluminum anti-size.

Rings at the end of every compression stage are presented in Figure 7.

In this figure, the dimensional changes of the ring for each lubricant during the three compression stages can be observed. These changes will be explained in detail in a subsequent section.

### 2.6. Finite Element Model Validation

**Figure 8:**
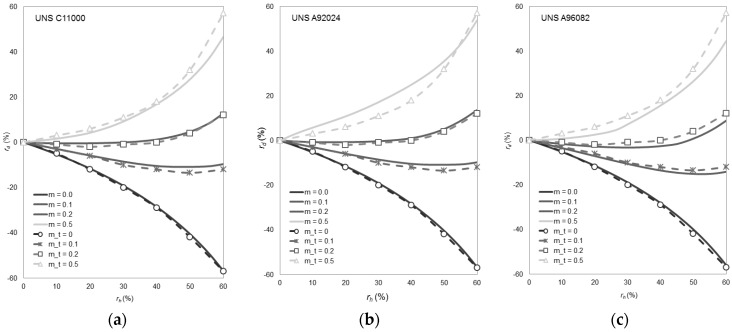
Finite element model validation with theoretical results obtained by De Pierre et al. [29]. (**a**) Copper alloy UNS C11000; (**b**) Aluminum alloy UNS A92024; (**c**) Aluminum alloy UNS A96082.

The finite element model has been validated with theoretical results obtained by De Pierre et al. [29] by an analytical method for the canonical aspect ratio (6:3:2). Due to the dependency of the method with the material, results for the same finite element model and different metallic alloys are presented in Figure 8. These alloys are (form left to right): copper alloy UNS C11000 and aluminum alloys UNS A92024 (both flow curves are imported from the materials library of DEFORM F2™) and UNS A96082 (used in this work).

**Figure 9:**
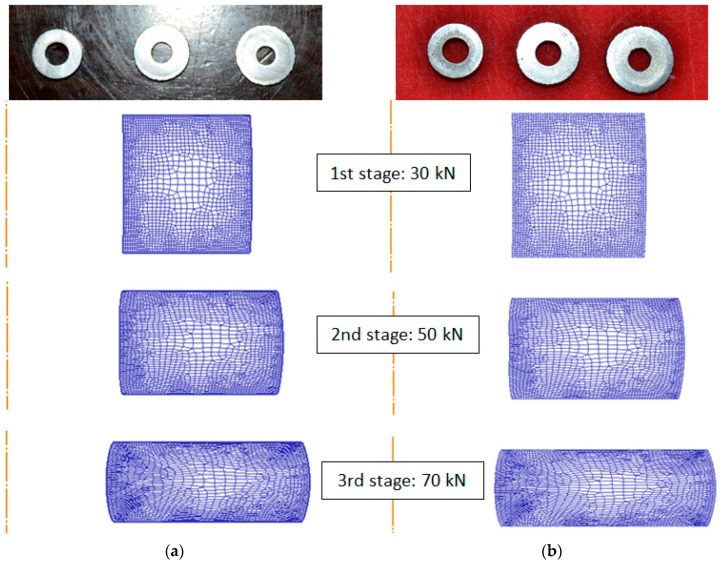
Comparison of experimental observations and numerical results. (**a**) Lubricant: silicone oil; (**b**) Lubricant: aluminum anti-size.

The biggest differences are found for the friction factor *m* = 0.5. However, these differences are minimum for the friction coefficients most commonly used in cold forming (m < 0.2). Additionally, a comparison of the dimensional changes in diameter obtained by experiments and finite element simulation is shown in Figure 9. In this figure, it can be seen graphically how the inner diameter decreases in both cases during the compression process (lubricants: silicone oil and aluminum anti-size).

Results from the simulation are in good agreement with both analytical and experimental results, so the numerical model can be considered validated.

## 3. Results and Discussion

### 3.1. Determination of Dimensional Variables

**Figure 10:**
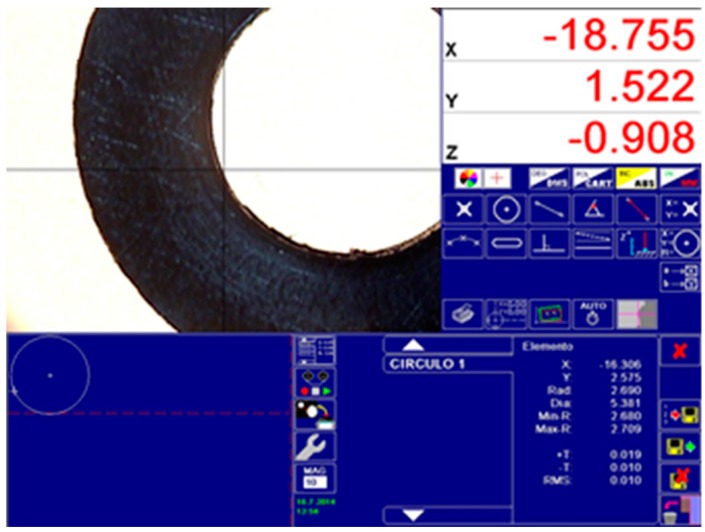
Details of the inner diameter measurement with profile projector TESA VISIO.

Once RCTs have been conducted in the laboratory, it is possible to create the friction factor map; to accomplish this, the reductions in height (Equation (5)) and inner radius (Equation (6)) that each ring experienced under different lubricants have to be obtained:

(5) $$r_{h}(\%)=\frac{h_{0}-h_{1}}{h_{0}}\cdot 100$$

(6) $$r_{d}(\%)=\left(\frac{d_{i,0}-d_{i,1}}{d_{i,0}}\right)\cdot 100$$

where *d*_i,0_ is the inner diameter of the ring at the beginning, *d*_i,1_ is the inner diameter of the ring at the end of the stage, *h*_0_ is the initial height of the ring, and *h*_1_ is the final height at every reduction stage. To measure the dimensional changes of the inner diameter, different methods have been proposed. Wang and Lenard [15] measured the diameter with calipers in two directions at the middle and on the end of the specimen. Hartley et al. [30] proposed a new method by using a ball-bearing of known diameter, and more recently, Shahriari et al. [19] combined the aforementioned method with a profile projector equipped with an X-Y micrometer table; however, this technique still offered problems because the method assumes the deformed profile possesses a circular shape which does not match perfectly to reality. Goetz et al. [31] demonstrated that there are more types of profiles besides concave and convex, which reduces the feasibility of the measurement process. For the sake of simplicity, and as there is no perfectly established procedure, a profile projector, TESA VISIO (TESA SA, Renens, Switzerland) (Figure 10), has been used in this paper to take the measurements of the middle plane.

Table 2 shows the measurements obtained after performing the RCT with each ring in every stage of the compression process. Each value of the diameter is the mean of three measurements, and each measurement is provided by the profile projector selecting eight points at 45° around the diameter; the diameter of the circle is determined by fitting the data by the least-square method.

As it can be seen in Table 2, the outer diameter expands in all cases; while the inner diameter increases at the first stage and decreases at the last stages during the compression process.

**Figure 11:**
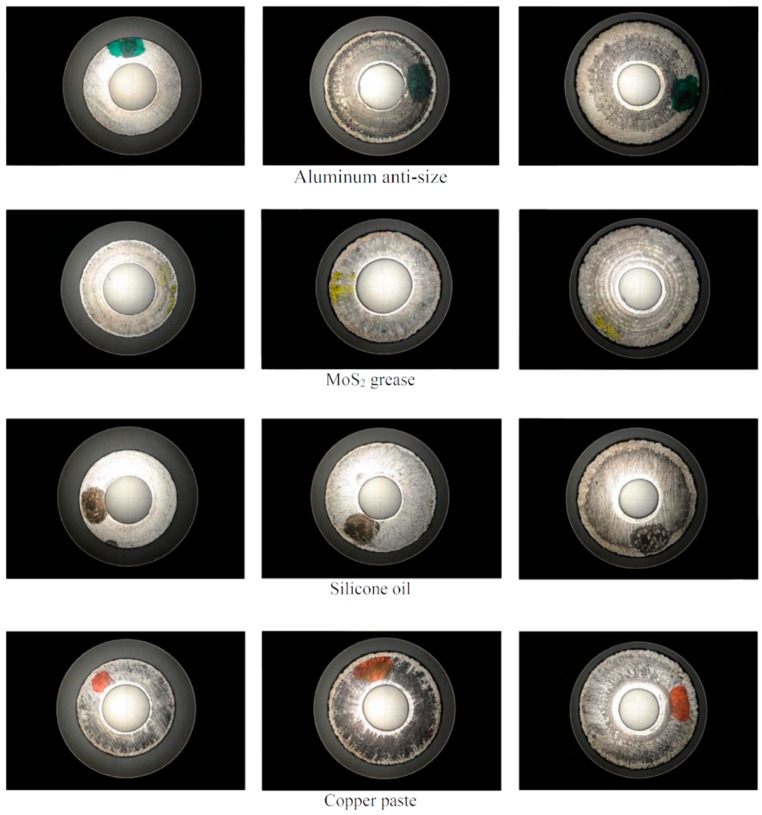
Rings tested in the laboratory after each forming stage. Images obtained by TESA VISIO profile projector.

Images (from the profile projector) of the rings’ surfaces that have been in contact with the flat platens for all lubricants are shown in Figure 11. The profile projector allows for the opportunity to see the contact surfaces of the workpiece after each compression stage. In all the cases there is an external ring belonging to the lateral surface of the ring that establishes new contact once the compression takes place; this will lead to an inhomogeneous contact surface and it will influence the friction factor so it can be one reason not to consider this factor *m* constant throughout the process.

### 3.2. Dual Friction Factor Map

**Figure 12:**
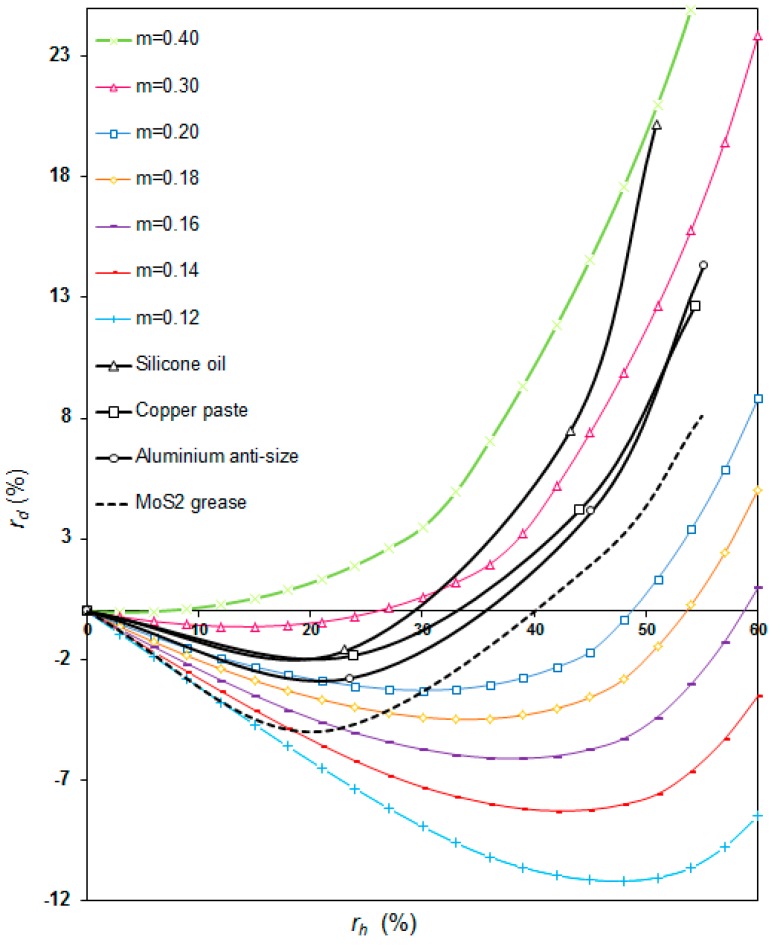
Dual friction factor map for UNS A96082 experimental results.

The friction factor map was obtained by a finite element model according to the material properties of the ring (aluminum alloy UNS A96082). By overlapping the friction curves obtained numerically and through experimental testing, a quantitative and qualitative assessment of the behavior of each lubricant can be realized, and some considerations about alternative uses of RCTs are described. Figure 12 shows the dual friction factor map obtained.

The dual friction factors map plotted for alloy UNS A96082 (Figure 12) shows that the value of the friction factor obtained experimentally for each lubricant can only be considered constant for small reductions in height (lower than 20% in most cases). Table 3 presents the friction factors estimated by comparing the experimental and simulated results. However, for reductions higher than this percentage, the assumption of a constant value for the friction coefficient throughout the RCT is no longer acceptable. Table 3 shows the ranges of friction factors encountered for each lubricant, and the average friction coefficient for each range.

**Figure 13:**
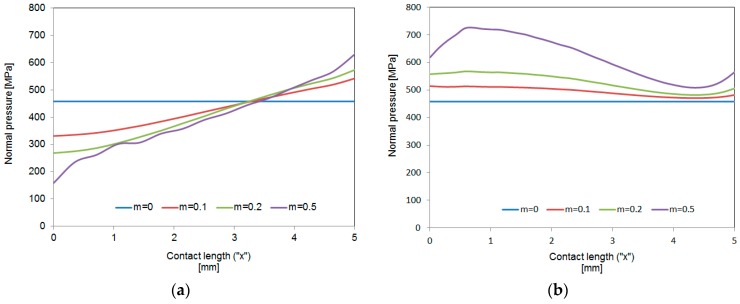
Contact pressure profiles for different friction conditions. (**a**) Slim preform (*d* = 10 mm and *h* = 20 mm); (**b**) Flattened-shape preform (*d* = 10 mm and *h* = 3 mm).

This behavior is reasonable because the tribological conditions are changing at the dies-workpiece interfaces during deformation caused by heavily loaded regimes, as observed in real transient metal forming operations, such as open die forging or stamping. On one hand, new contact areas corresponding to the lateral surfaces of the ring are establishing new contact, and, on the other hand, the lubricant layer during large height reductions is not expected to be homogeneously distributed at the contact area according to the inhomogeneous contact pressures profile [32]. As an example, Figure 13a shows the contact pressure profile for a slim preform, as it occurs in the early stages of open die forging of cylindrical components, and Figure 13b presents the same profile for a flattened-shape preform, as in the late stages during compression of cylindrical parts.

As observed in the first case (Figure 13a), a minimum is detected at the center; this configuration can be favorable to entrap the lubricant at this area, resulting in positive response against friction. However, in the second case (Figure 13b) the maximum of the profile is presented around the center of the workpiece, as expected from classical analysis [33]; under this configuration the lubricant layer at the center could break and slip towards the edge of the workpiece. In both cases, every contact pressure profile has a different friction condition associated with it and vice versa. This means that during any transient metal forming operation, such as forging, tribological conditions change during the process as a result of different stress states, so the establishment of the initial friction factor for the entire operation, as usual, should be reconsidered.

Furthermore, in metal forming processes several types of lubrication can be distinguished [27]. In dry forming there is no lubricant at the interface so friction is high. In boundary lubrication there is a thin film of lubricant, generally organic, that is physically absorbed or chemically adhered to the material surface. As in the former case, friction remains high. Full film lubrication occurs when there is a thick layer of solid lubricant; in this case, the conditions affecting friction are governed by the shear strength of the lubricant film. When there is a thick layer of liquid lubricant, the lubrication conditions are hydrodynamic; in this case, friction is governed by the viscosity of the lubricant and the sliding velocity at the contact interface allowing friction to be considered relatively low. However, the most common situation in metal forming operations is mixed-layer lubrication; this is because hydrodynamic conditions cannot be maintained at high pressures and low sliding velocities. Taking this into account, it is necessary to establish a new procedure to determine conditions of friction in metal forming processes closer to those actually encountered.

As indicated by Mandić and Stefanović [28], modelling results and their transfer onto real processes highly depends upon the similarity of the contact friction conditions in modelling and during the real process. According to this, the friction factor at the very beginning of any forming process, when friction conditions can be significantly different to the ones resulting at the end of the process, can be far from reality. For small height reductions (lower than 20% in the case of the material considered in this paper, UNS A96082) friction curves obtained experimentally for each lubricant are clearly similar to some of the curves obtained by the finite element simulation. However, for high height reductions (higher than 20% in the case of UNS A96082), this behavior is not observed, and the experimental results fall into a friction factor range for each lubricant. In this case, the use of a single average value of the friction factor assigned to every lubricant-component pair in the range of deformation is recommended.

## 4. Conclusions

This paper shows that friction factors obtained for each lubricant-surface pair by the alternative approach of the RCT technique seem to vary during the compression process, as observed in real transient metal forming operations; thus, the points obtained do not entirely match a single curve of the friction factor map plotted from the finite element simulation.

Based on these observations, the alternative approach of the RCT presented in this paper can be successfully applied in the determination of friction conditions that are better adapted to real processes. To obtain a friction factor closer to the actual one present in real forming processes, the new procedure described in this paper is recommended:

- For small deformation stages (*r_h_* < 20% in this work), the conventional RCT [10] can be applied, as the tribological conditions will remain similar during the operation (Table 3). Particularly, in this paper, friction factors obtained for small reductions were 0.12, 0.20, 0.23, and 0.24 for lubricants MoS_2_, aluminum anti-size, copper paste, and silicone oil, respectively.
- For large deformations-reductions (*r_h_* > 20% in this work), it would be advisable to use the alternative approach of the RCT presented in this paper (without any lubrication between steps), and to obtain an average value of the friction factor assigned to every lubricant-component pair in the range of deformation considered (Table 3). This average value was 0.18, 0.24, 0.25, and 0.32 for lubricants MoS_2_, aluminum anti-size, copper paste, and silicone oil, respectively. Some differences were also observed in the width of the range obtained for each lubricant, being small for aluminum anti-size and copper paste (0.08 and 0.04, respectively) and bigger for MoS_2_ and silicone oil (0.12 and 0.15, respectively). In these last two cases the determination of an average value is more critical.

The methodology presented in this paper can be used in the real practice of forming processes as follows. In incremental processes and those ones where lubrication can be applied between stages of small deformations, the conventional RCT is advisable for taking into account that friction conditions are more homogeneous with the application of incremental loads [34]. However, in those metal forming operations where heavy loaded regimes occur and the lubricant cannot be applied in intermediate stages, the lubricant layer is not constant during the forming process, and the tribological conditions change throughout the operation. When a transient forming process, such as forging or stamping, is being performed, the use of the alternative RCT approach is recommended. In this case, an average value for the whole friction range should be estimated instead of applying the initial friction factor for the entire operation, as usual. For more precise results, the friction factor should be selected according to the deformation stage in each particular case. Thus, the friction factor values should be based on the deformation level of each particular forming process, and this alternative approach of the RCT can be valuable.

To help in this matter, dual friction factor maps, as presented in this work, can be an interesting tool to determine friction coefficients at die-workpiece interfaces closer to those found in real process conditions.

## Figures and Tables

**Table 1:** Ring compression test conditions.

Temperature (°C)	Ram Velocity (mm/min)	1st Stage Load (kN)	2nd Stage Load (kN)	3rd Stage Load (kN)
20	2.5	30	50	70

**Table 2:** Dimensional measurements after conducting the RCTs.

Lubricants	Forming Stage	Inner Diameter (mm)	Outer Diameter (mm)
Aluminum anti-size	1	5.149	10.989
	2	4.803	12.510
	3	4.294	13.680
MoS_2_ grease	1	5.259	11.080
	2	4.878	12.754
	3	4.476	13.735
Silicone oil	1	5.093	10.842
	2	4.638	12.383
	3	4.002	12.982
Copper paste	1	5.102	10.943
	2	4.800	12.538
	3	4.377	13.598

**Table 3:** Friction factors determined by the RCT according to reduction in height.

Friction Factor (*m*)	MoS_2_ Grease	Aluminum Anti-Size	Copper Paste	Silicone Oil
Small height reduction (*r_h_* < 20%)	0.12	0.20	0.23	0.24
Large height reduction (*r_h_* > 20%) *	0.12 < *m* < 0.24, *m*_avg_ = 0.18	0.20 < *m* < 0.28, *m*_avg_ = 0.24	0.23 < *m* < 0.27, *m*_avg_ = 0.25	0.24 < *m* < 0.39, *m*_avg_ = 0.32

* *Upper value: friction coefficients range; lower value: average friction coefficient.*
